# The Joint Effects of Diet and Dietary Supplements in Relation to Obesity and Cardiovascular Disease over a 10-Year Follow-Up: A Longitudinal Study of 69,990 Participants in Australia

**DOI:** 10.3390/nu13030944

**Published:** 2021-03-15

**Authors:** Xiaoyue Xu, Zumin Shi, Gang Liu, Dennis Chang, Sally C. Inglis, John J. Hall, Aletta E. Schutte, Julie E. Byles, Deborah Parker

**Affiliations:** 1 School of Population Health, University of New South Wales, Sydney, NSW 2052, Australia; john.hall@unsw.edu.au (J.J.H.); a.schutte@unsw.edu.au (A.E.S.); 2 The George Institute for Global Health, Sydney, NSW 2042, Australia; 3 Improving Palliative, Aged and Chronic Care through Clinical Research and Translation Research Centre, Faculty of Health, University of Technology Sydney, Sydney, NSW 2007, Australia; sally.inglis@uts.edu.au (S.C.I.); Deborah.Parker@uts.edu.au (D.P.); 4 Human Nutrition Department, College of Health Sciences, QU Health, Qatar University, Doha 2713, Qatar; zumin@qu.edu.qa; 5 Faculty of Science, School of Life Sciences, University of Technology Sydney, Sydney, NSW 2007, Australia; Gang.Liu@uts.edu.au; 6 Centre for Inflammation, Centenary Institute, Sydney, NSW 2050, Australia; 7 NICM Health Research Institute, Western Sydney University, Westmead, NSW 2145, Australia; d.chang@westernsydney.edu.au; 8 Hypertension in Africa Research Team, Medical Research Council Unit for Hypertension and Cardiovascular Disease, North-West University, Potchefstroom 2520, South Africa; 9 Centre for Women’s Health Research, School of Medicine and Public Health, University of Newcastle, Newcastle, NSW 2308, Australia; julie.byles@newcastle.edu.au

**Keywords:** multivitamins and minerals, fish oil, calcium, dietary, cardiovascular health

## Abstract

It is unknown whether a healthy diet or unhealthy diet combined with specific supplements may jointly contribute to incidence of obesity and cardiovascular disease (CVD). We included 69,990 participants from the 45 and Up Study who completed both baseline (2006–2009) and follow-up (2012–2015) surveys. We found that compared to participants with a long-term healthy diet and no supplement consumption, those with a long-term healthy diet combined with multivitamins and minerals (MVM) or fish oil consumption were associated with a lower incidence of CVD (*p* < 0.001); whilst those with an unhealthy diet and no MVM or fish oil consumption were associated with a higher risk of obesity (*p* < 0.05). Compared to participants with a long-term healthy diet and no calcium consumption, the combination of a long-term healthy diet and calcium consumption was linked to a lower risk of CVD (IRR = 0.87, 95% CI: 0.78; 0.96). In conclusion, a long-term healthy diet combined with MVM or fish oil was associated with a lower incidence of CVD. Participants who maintained a healthy diet and used calcium supplements were associated with a lower incidence of obesity. However, these associations were not found among those with an unhealthy diet, despite taking similar supplements.

## 1. Introduction

Diet is one of the most important modifiable factors in the prevention of chronic diseases, including obesity and cardiovascular disease (CVD) [1]. Diets rich in fruit and vegetable reduce the risk of coronary heart disease [2]. Fish are rich in anti-oxidative omega-3 fatty acids that can attenuate cerebrovascular disease [3,4]. High-protein diets are associated with lowering of obesity, as well as lower blood pressure and triglyceride levels [5].

People consume nutritional supplements when they believe there are nutritional gaps in their diets where these supplements may help prevent diseases [6], but the scientific evidence supporting dietary supplements use is mixed [7,8,9]. For example, some large-scale, randomized controlled trials suggest use of multivitamins and mineral (MVM) supplements reduce the risk of CVD and reduce body mass index (BMI) [10]. However, a systematic review and meta-analysis of 18 studies with 2,019,862 participants found no association between MVM and mortality from CVD, coronary heart disease and stroke, or stroke incidence [11]. Although a significant number of studies has reported the association between calcium supplementation and adverse cardiovascular events [12], Chung et al.’s systematic review of 31 studies concluded that calcium intake within 2000 to 2500 mg/d (from either food or supplement sources) is not associated with CVD risk [13]. 

In addition, it is unknown whether a healthy diet combined with specific supplements may jointly contribute to better health, or whether dietary supplements combined with a poor diet may yield some additional protection against obesity or CVD. Moreover, as it has been widely reported that obesity is associated with numerous comorbidities such as CVD [14], we examined the joint effects of diet and supplements in relation to people with both obesity and CVD. Our study aimed to explore the joint effects of diet and dietary supplements in relation to obesity and CVD with the hypothesis that people who had healthy diet and dietary supplements may achieve better health outcomes. The specific research aims were to examine (1) whether diet and individual dietary supplements are related to the incidence of CVD, obesity, or both; and (2) if the joint effects of long-term diet and dietary supplements are related to the incidence of CVD, obesity, or both. We chose MVM, fish oil, and calcium as the supplements of interest given that (1) they are the most commonly used supplements within the Australian population, and (2) they are the most controversial in terms of the relationship to disease outcomes.

## 2. Materials and Methods

### 2.1. Study Design

We analyzed baseline (2006–2009) and follow up (2012–2015) data from the 45 and Up Study. Participants were randomly sampled from the Department of Human Services enrolment database through which national healthcare is administered and which includes all citizen and permanent residents of Australia, as well as some temporary residents and refugees [15,16]. A total of 267,153 men and women aged 45 and over across New South Wales, Australia, were recruited and surveyed in 2006–2009, representing about 10% of this age group. People aged 80 years and over and people living in rural areas were oversampled, and the sample also skewed to higher socioeconomic status. Upon recruitment, participants provided consent for future follow-up. The first follow-up survey data were collected between 2012 and 2015. At both time points, socioeconomic, health behavior and health-related information were collected via a comprehensive questionnaire. Details of the 45 and Up Study, including sampling strategy, are described elsewhere [16]. The baseline and follow-up questionnaires are available at the Sax Institute website (https://www.saxinstitute.org.au/our-work/45-up-study/questionnaires/, (accessed on 15 July 2019)).

The conduct of the 45 and Up Study, including participants’ informed consent, was approved by the University of New South Wales Human Research Ethics Committee. Analysis of the 45 and Up Study for the present study was approved by The University of Technology, Sydney (ETH18-2145).

**Figure 1:**
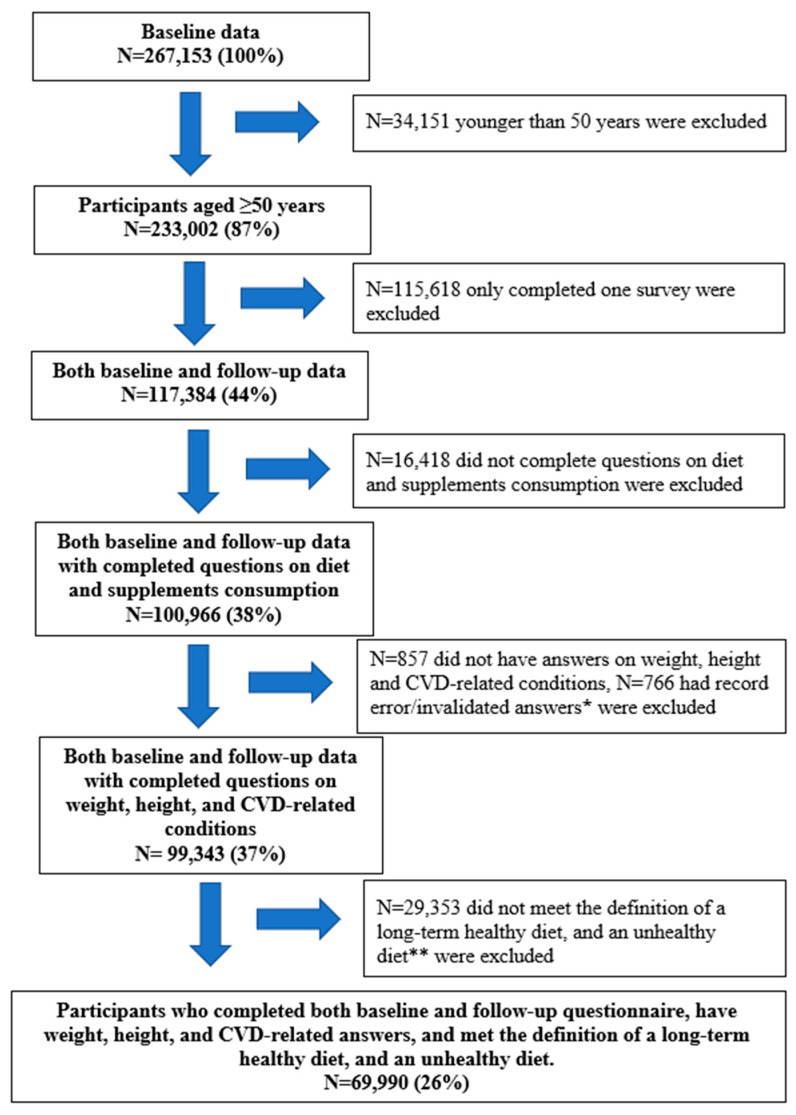
Study flow chart. * People who reported having cardiovascular disease (CVD)/obesity at baseline but had no CVD/obesity at follow-up. ** People who had a healthy diet/an unhealthy diet at baseline but had an unhealthy diet/a healthy diet, respectively, at follow-up.

In the present study, we included participants (N = 69,990) who completed both baseline and follow-up questionnaires, were at least 50 years old, had weight, height, and CVD-related answers, and met the definition of a long-term healthy or unhealthy diet. Figure 1 presents the study flow and the sample size at each stage of exclusion, including initially a total of 233,022 participants aged 50+ who provided the baseline data (87.2% of the total population) and 142,010 participants who provided the follow-up data (53.2%). 

### 2.2. Outcomes

Participants were identified as having CVD (including CVD-related conditions) at baseline and follow-up separately if they reported to have (1) physician-diagnosed thrombosis, heart failure, atrial fibrillation, heart disease, or stroke, and/or (2) recent treatment (in the last month) for thrombosis, heart attack or angina, or other types of heart disease. 

BMI was calculated based on the formula of weight in kilograms over height in meters squared. Weight and height were self-reported at baseline and follow-up based on the questions, “about how much do you weigh?” and “how tall are you without shoes?”. Obesity was defined as BMI greater than or equal to 30.0 kg/m^2^ using the World Health Organization criteria. 

We focused on three study outcomes in this study: (1) participants with CVD, (2) participants with obesity, and (3) participants with both CVD and obesity.

### 2.3. Long-Term Dietary Consumption

Based on the Australian Dietary Guideline (ADG), the seven food components of interest included vegetables, fruit, grains, lean meat and poultry and seafood, dairy, food diversity, and alcohol consumption [17,18]. In the 45 and Up questionnaire, dietary consumption was assessed by short food frequency questions, which have been described in previous research [19,20]. Each of the questions on diet were previously validated in the Million Women Study [21]. 

Adequate fruit (≥2 servings per day) and vegetable (≥5.5 servings per day for men aged 51-70 years, ≥5 servings per day for men aged 70+ years and for women across all age groups) consumption was identified according to the ADG. The frequency of food groups of grains (low: 0–4, medium: 4–7, and high: >7 times per week) and “lean meat, poultry and seafood” (low: 0–6, medium: 6–8, and high: >8 times per week) was divided into three quantiles indicating consumption from low to high. Dairy was categorized as Yes/No. Food diversity was identified if participants consumed all five food groups mentioned above. Harmful drinking was identified as >10 standard drinks (a glass of wine, middy of beer, or nip of spirits) per week [18].

We created dietary consumption scores based on seven food components from ADG to capture the overall dietary consumption. The score range was from 0 (the healthiest dietary behavior) to 9 (the unhealthiest behavior) (Table S1). We further identified dietary consumption as healthy diet (lower than mean) and unhealthy diet (higher than mean). A long-term healthy diet was identified if participants met the criteria for a healthy diet for both waves.

### 2.4. Dietary Supplements

Dietary supplements of interest included MVM, fish oil omega-3, and calcium. Participants were defined as consuming supplements if they answered “yes” to the question “Have you taken multivitamins and minerals/fish oil omega-3/calcium in the past 4 weeks?”. For each supplement, we created four subgroups according to answers from both baseline and follow-up surveys: (a) no supplement consumption at baseline and follow-up, (b) no supplements at baseline but had supplement consumption at follow-up, (c) supplements at baseline but no supplement consumption at follow-up, and (d) supplement consumption at baseline and follow-up.

### 2.5. Joint Effects of Diet and Supplements

We examined the joint effects of diet (healthy/unhealthy) and supplements (four subgroups of MVM, fish oil, and calcium respectively) on the incidence of CVD, obesity, or both. Group classifications are shown in Table S2. We treated the “healthy diet and no supplements” as the reference group for further analysis based on the hypothesis that people can get enough nutrients from healthy food consumption. 

### 2.6. Covariates

We included socio-demographic factors and health behavioral factors as covariates in the analysis. Socio-demographic variables included age, country of birth, marital status, education, and socioeconomic level [22], and health behaviors included smoking and physical activity levels. Country of birth was categorized as Australian versus other countries. Marital status was categorized as married/partner, single/divorce/separated, and widowed. Education levels were divided into three categories: Low, no school certificate or other qualification, and school, or intermediate certificate; Medium, high school or leaving certificate, and trade or apprenticeship; and High: certificate or diploma, and university degree or higher. Socioeconomic levels were assessed by Socio-Economic Indexes for Areas (SEIFA), which is based on three quantiles (low, medium, high) of Index of Relative Socio-economic Advantage and Disadvantage [19]. 

Smoking was identified as never smoked, previous smoker, and current smoker, based on two questions: “Have you ever been a regular smoker?”, and “Are you a regular smoker now?”. Physical activity was measured using the Active Australia Survey, asking the total time spent on walking, and on moderate-intensity and vigorous-intensity physical activity in the previous week. Adequate physical activity was identified if people spent 150 minutes of moderate intensity physical activity, or 75 minutes of vigorous intensity physical activity per week [23,24]. 

We also included self-reported treatment for dyslipidemia, diagnosed hypertension, and diabetes as covariates given the association of these conditions with CVD and obesity. 

### 2.7. Statistical Analysis

**Figure 2:**
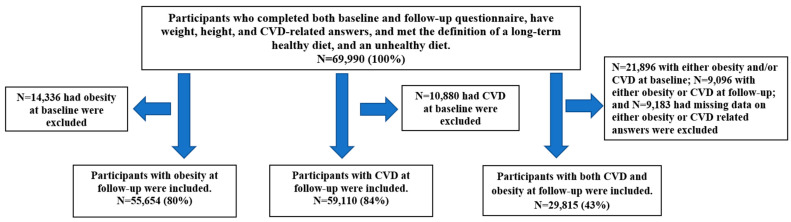
Flow chart of numbers of participants with data on obesity and CVD.

Participants’ characteristics were compared between baseline and follow-up using chi-square tests. Poisson regression models with robust variance [25] were applied to assess (1) diet and the consumption of each supplement in relation to the incidence of CVD, obesity, or both; and (2) the joint effects of long-term diet and supplements consumption in relation to the incidence of CVD, obesity, or both. The results were reported in tables or forest plots, with incidence rate ratios (IRR) and 95% confidence intervals (CI) in four models, i.e., crude model; model 1 after adjustment for socioeconomic factors; model 2 after adjustment for health behavior factors and model 1; and model 3 after adjustment for chronic conditions and model 2. The number of participants included in the analysis is shown in Figure 2.

Because we excluded participants who had obesity and/or CVD at baseline, there may have been a bias towards a higher incidence of outcomes. Therefore, we conducted sensitivity analysis by including participants with obesity and/or CVD at baseline and assumed this remained at follow-up (N = 69,990). Poisson regression models with robust variance were performed in the sensitivity analysis. 

A post hoc analysis using a generalized estimating equation model was applied allowing diet and dietary supplements variables to vary between baseline and follow-up. Of a total of 100,109 participants, we classified four joint subgroups for each supplement at baseline and follow-up separately, namely (a) healthy diet (+), no MVM (–); (b) healthy diet (+), had MVM (–); (c) unhealthy diet (–), no MVM (–); and (d) unhealthy diet (–), had MVM (–); and we tested its relationship to obesity and CVD. Odds ratios (OR) and 95% CI were reported in tables or forest plots. All analyses were conducted in STATA/SE 14 (StataCorp, College Station, TX, USA).

## 3. Results

### 3.1. Participant Characteristics 

Participant characteristics at baseline and follow-up are described in Table 1. Significant differences in age, marital status, and socioeconomic levels were observed. Fish oil was the most commonly used supplement, followed by MVM and calcium. There were 26.9% of participants in the obese BMI range at baseline and 35.2% at follow-up; 15.6% had CVD at baseline and 29.2% at follow-up; 5.6% at baseline had comorbid obesity and CVD and 12.0% at follow-up. 

Among participants who changed diet (from healthy to unhealthy) from baseline to follow-up, 68.4% were aged 50–71 years, 53.8% were women, 74.8% were married, 34.2% lived in high SEIFA, and 27.1% had high education levels.

### 3.2. Diet, Dietary Supplements, and Obesity and/or CVD

As shown in Table 2, participants with an unhealthy diet had a higher risk of obesity (IRR = 1.10, 95% CI: 1.02; 1.18, model 4) compared with those with a long-term healthy diet. No significant association were found between diet and CVD, and comorbid obesity and CVD after adjusted for socioeconomic factors, health behavior factors, and other chronic conditions. MVM was inversely associated with the incidence of CVD in the crude model (IRR = 0.81, 95% CI: 0.77; 0.85), IRR of 0.92 (95% CI: 0.87; 0.96) in model 3, and IRR of 0.94 (95% CI: 0.89; 0.99) in model 4. MVM was also inversely associated with the incidence of comorbid obesity and CVD in the crude model (IRR = 0.69, 95% CI: 0.55; 0.85), IRR of 0.78 (95% CI: 0.62; 0.97) in model 2, and IRR of 0.79 (95% CI: 0.64; 0.99) in model 3.

No significant associations were found between changed diet from baseline to follow-up and CVD. However, compared with participants who had healthy diet at both waves, participants who changed their diet (from healthy to unhealthy) had an IRR of 1.07 (95% CI: 1.04; 1.10) for obesity.

Fish oil was inversely associated with the incidence of CVD across all models. Fish oil was also inversely associated with the incidence of comorbid obesity and CVD in the crude model (IRR = 0.75, 95% CI: 0.63; 0.91) and in model 2 (IRR = 0.80, 95% CI: 0.66; 0.98).

Calcium was inversely associated with the incidence of obesity (IRR = 0.83, 95% CI: 0.75; 0.91, model 4). However, calcium was positively associated with the incidence of CVD in the crude model (IRR = 1.07, 95% CI: 1.01; 1.13) and in model 2 (IRR = 1.09, 95% CI: 1.02; 1.15). 

### 3.3. The Joint Effects of Diet and Supplements in Relation to Obesity and/or CVD

**Figure 3:**
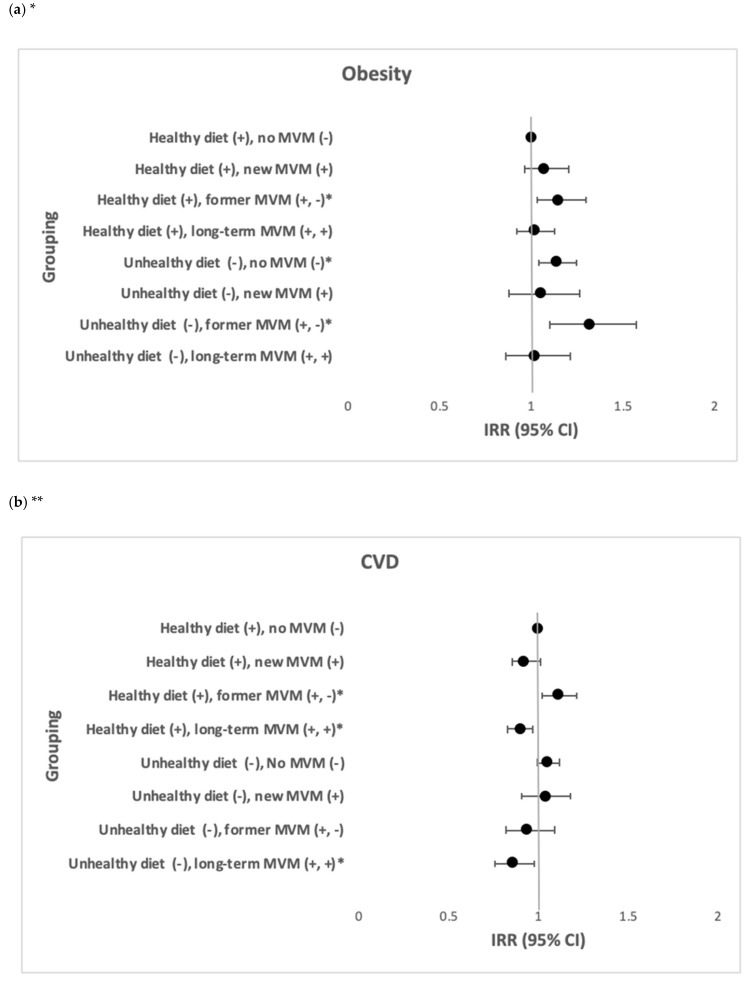
The joint effects of healthy vs. unhealthy diet and minerals and multivitamins. consumption in relation to the incidence of obesity and CVD (N = 69,990) (**a**) The joint effects of healthy vs. unhealthy diet and minerals and multivitamins. consumption and in relation to the incidence of obesity. (**b**) The joint effects of healthy vs. unhealthy diet and minerals and multivitamins. consumption and in relation to the incidence of CVD. * IRR after adjustment of age, gender, country of birth, marital status, education, SEIFA, smoking, physical activity, CVD, diabetes, blood cholesterol and blood pressure. ** IRR after adjustment of age, gender, country of birth, marital status, education, SEIFA, smoking, physical activity, obesity, diabetes, blood cholesterol and blood pressure.

Among participants with a healthy diet, those with former MVM consumption had a higher risk of obesity (IRR = 1.15, 95% CI: 1.03; 1.30) and CVD (IRR = 1.11, 95% CI: 1.02; 1.21), whilst those with a long-term MVM consumption had a lower risk of CVD (IRR = 0.90, 95% CI: 0.83; 0.97). Among participants with an unhealthy diet, those with no MVM consumption (IRR = 1.14, 95% CI: 1.04; 1.25) and those with former MVM consumption (IRR = 1.32, 95% CI: 1.10; 1.57) had a higher risk of obesity, whist those with a long-term MVM consumption had a lower risk of CVD (IRR = 0.86, 95% CI: 0.76; 0.98) (Figure 3). Participants with a healthy diet and long-term MVM consumption had a lower risk of comorbid obesity and CVD across all four models (Table 3).

**Figure 4:**
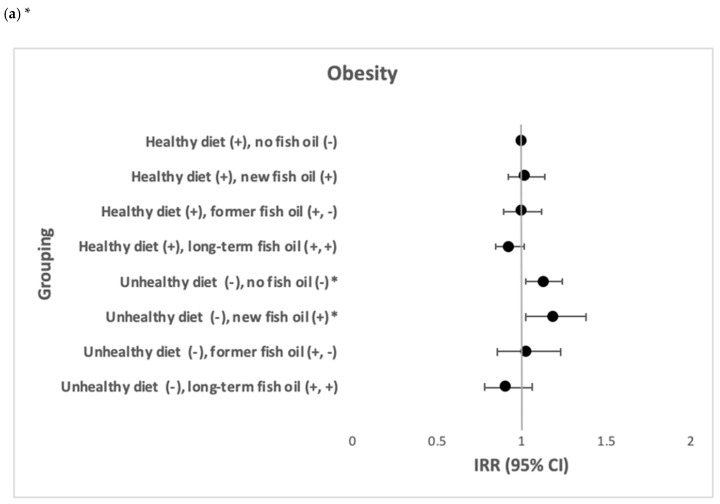

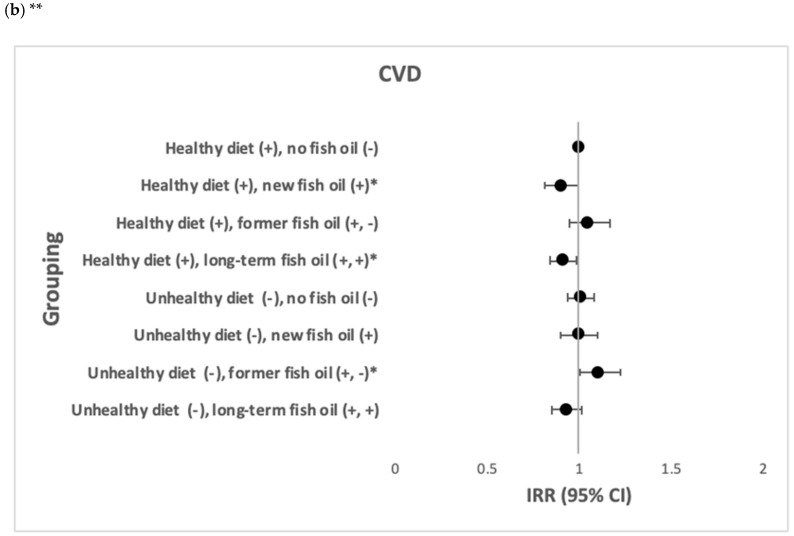
The joint effects of healthy vs. unhealthy diet and fish oil consumption in relation to the incidence of obesity and CVD (N = 69,990). (**a**) The joint effects of healthy vs. unhealthy diet and fish oil consumption in relation to the incidence of obesity. (**b**) The joint effects of healthy vs. unhealthy diet and fish oil consumption in relation to the incidence of CVD. * IRR after adjustment of age, gender, country of birth, marital status, education, SEIFA, smoking, physical activity, CVD, diabetes, blood cholesterol and blood pressure. ** IRR after adjustment of age, gender, country of birth, marital status, education, SEIFA, smoking, physical activity, obesity, diabetes, blood cholesterol and blood pressure.

No joint effects of healthy diet and fish oil consumption in relation to obesity were found. Among participants with a healthy diet, those with new fish oil consumption (IRR = 0.91, 95% CI: 0.84; 0.99) and those with a long-term fish oil consumption (IRR = 0.90, 95% CI: 0.85; 0.96) had a lower risk of CVD. Among participants with a long-term unhealthy diet, those with no fish oil consumption (IRR = 1.13, 95% CI: 1.02; 1.24) and those with new fish oil consumption had a higher risk of obesity (IRR = 1.19, 95% CI: 1.02; 1.38), whereas those with former fish oil intake (IRR = 1.10, 95% CI: 1.00; 1.22) had a higher risk of CVD (Figure 4).

Participants with a healthy diet and new fish oil consumption (IRR = 0.70, 95% CI: 0.50; 0.98, model 2) and those with long-term fish oil consumption (IRR = 0.72, 95% CI: 0.54; 0.96, model 3) had a lower risk of comorbid CVD and obesity. Participants with an unhealthy diet and no fish oil consumption also had a lower risk of comorbid CVD and obesity in the crude model (IRR = 0.63, 95% CI: 0.40; 0.98) (Table 3). 

**Figure 5:**
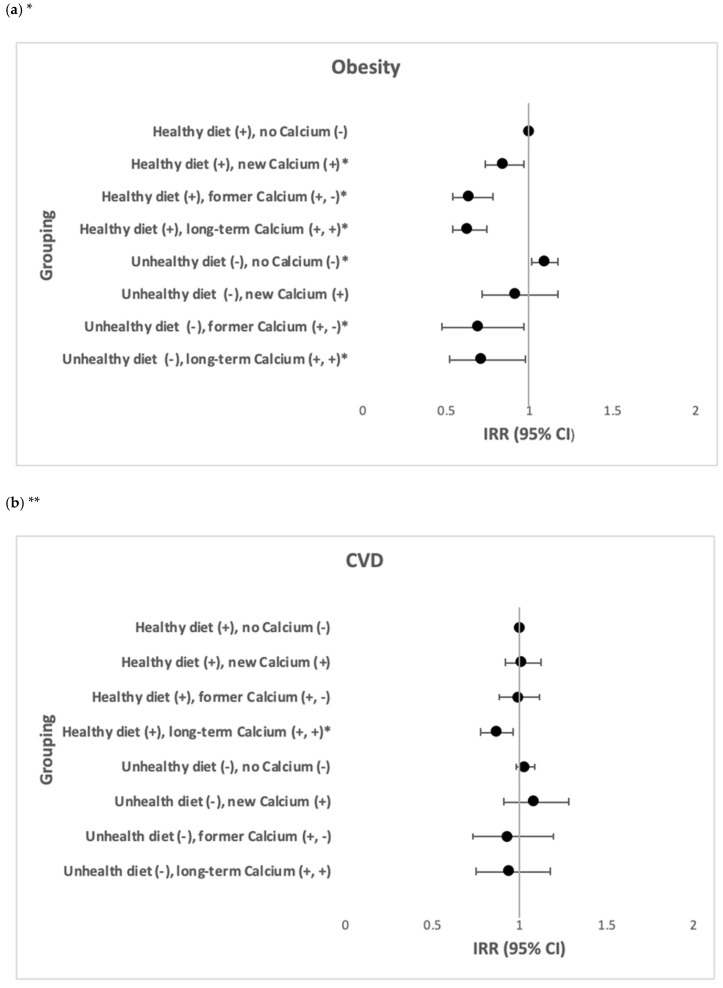
The joint effects of healthy vs. unhealthy diet and calcium consumption in relation to the incidence of obesity and CVD (N = 69,990) *. (**a**) The joint effects of healthy vs. unhealthy diet and calcium consumption in relation to the incidence of obesity. (**b**) The joint effects of healthy vs. unhealthy diet and calcium consumption in relation to the incidence of CVD. * IRR after adjustment of age, gender, country of birth, marital status, education, SEIFA, smoking, physical activity, CVD, diabetes, blood cholesterol and blood pressure. ** IRR after adjustment of age, gender, country of birth, marital status, education, SEIFA, smoking, physical activity, obesity, diabetes, blood cholesterol and blood pressure.

Among participants with a healthy diet, those with new calcium consumption (IRR = 0.84, 95% CI: 0.73; 0.97) and those with former calcium consumption (IRR = 0.64, 95% CI: 0.54; 0.74) had a lower risk of obesity. Participants with a long-term calcium consumption had a lower risk of obesity (IRR = 0.63, 95% CI: 0.54; 0.74) and CVD (IRR = 0.87, 95% CI: 0.78; 0.96). Among participants with an unhealthy diet, those with no calcium consumption had a higher risk of obesity (IRR = 1.09, 95% CI: 1.01; 1.17); however, those with former calcium consumption (IRR = 0.69, 95% CI: 0.47; 0.97) and those with a long-term calcium consumption (IRR = 0.71, 95% CI: 0.52; 0.98) had a lower risk of obesity (Figure 5). No associations were observed between joint effect of diet and calcium and the incidence of comorbid obesity and CVD (Table 3).

### 3.4. Sensitivity Analysis

A total of 69,990 participants were included in the sensitivity analysis (Figures S1–S3, Table S3). Compared with our previous analysis, consistent results were found as described below. Among participants with a healthy diet, those with former MVM consumption had a higher risk of obesity (IRR = 1.04; 95% CI: 1.01; 1.07), those with a long-term MVM consumption had a lower risk of CVD (IRR = 0.89, 95% CI: 0.86; 0.92) (Figure S1) and comorbid obesity and CVD (IRR = 0.85; 95% CI: 0.80; 0.91, model 4, Table S3). Among participants with an unhealthy diet, those with no MVM consumption (IRR = 1.03, 95% CI: 1.01; 1.06) and those with former MVM consumption had a higher risk of obesity (IRR = 1.07, 95% CI: 1.02; 1.12) (Figure S1). 

Among participants with a healthy diet, those with new fish oil consumption (IRR = 0.86, 95% CI: 0.83; 0.89) had a lower risk of CVD, and those with a long-term fish oil consumption had a lower risk of CVD (IRR = 0.91, 95% CI: 0.89; 0.94) (Figure S2) and comorbid obesity and CVD (Table S3).

Among participants with a healthy diet, those with new calcium consumption (IRR = 0.87, 95% CI: 0.84; 0.91), those with former calcium consumption (IRR = 0.80; 95% CI: 0.76; 0.84), and those with a long-term calcium consumption (IRR = 0.72; 95% CI: 0.69; 0.76) had a lower risk of obesity. Among participants with an unhealthy diet, those with former calcium consumption (IRR = 0.81, 95% CI: 0.74; 0.90), and those with a long-term calcium consumption (IRR = 0.69, 95% CI: 0.62; 0.76) had a lower risk of obesity (Figure S3).

In the sensitivity analysis, we found participants with a healthy diet and long-term fish oil had a lower risk of obesity; in addition, participants with unhealthy diet but with long-term fish oil had a lower risk of CVD (Figure S2). We also found that, compared with participants with healthy diet and no calcium consumption, those with either healthy or unhealthy diet combined with calcium consumption (including new, former, and long-term) were associated with lower risk of comorbid obesity and CVD (Table S3).

### 3.5. Post hoc Analysis 

A total of 100,109 participants were included in the post hoc analysis (Figures S4–S6, Table S4). Participants with a healthy diet and MVM consumption had a lower risk of CVD (OR = 0.89, 95% CI: 0.86; 0.92) and comorbid obesity and CVD, whilst those with an unhealthy diet and no MVM consumption had a higher risk of obesity (OR = 1.03, 95% CI: 1.01; 1.07) (Figure S4, Table S4). Participants with a healthy diet and fish oil consumption had a lower risk of CVD (OR = 0.95, 95% CI: 0.93; 0.98) and comorbid obesity and CVD (Figure S5, Table S4). Participants with a healthy diet and who had calcium consumption had a lower risk of obesity (OR = 0.80, 95% CI: 0.77; 0.84), and those with an unhealthy diet and no calcium consumption had a higher risk of CVD (OR = 1.04, 95% CI: 1.01; 1.06) (Figure S6).

## 4. Discussion

In a large population sample of middle-aged and older adults, we found that participants who maintained a healthy diet over the long term, combined with multivitamins and minerals or fish oil supplements, had a lower incidence of CVD than those not taking supplements. These associations were also found among participants with both CVD and obesity. Our results highlight that those who maintained a healthy diet combined with calcium supplements had a lower incidence of obesity. All of these associations were not evident in those with an unhealthy diet, despite taking similar supplements. 

Our findings show the benefit of multivitamins and minerals, particularly combined with a long-term healthy diet, in relation to the development of CVD. Epidemiological studies have widely indicated the importance of healthy eating in CVD prevention, but consistent results of the association between multivitamins and minerals and CVD have not been established [26,27]. A study including 18,530 male physicians from the Physicians’ Health Study also showed that multivitamins and minerals consumption for more than 20 years was associated with a lower risk of major CVD events [26], in contrast to a prospective cohort study in 37,197 women from the Women’s Health Study showing that multivitamin and mineral consumption was not associated with myocardial infarction, stroke, cardiac revascularizations, or CVD death [27]. Epidemiological studies examining the joint effect of diet and multivitamins and minerals in relation to comorbidities are scarce. 

We also found the benefit of fish oil, and also a long-term healthy diet combined with fish oil, to be associated with a lower risk of CVD. Previous studies raised much interest in the role of fish oil omega-3 in CVD prevention because of the effects of alpha-linolenic acid, eicosapentaenoic acid (EPA), and docosahexaenoic acid (DHA) contained in fish oil [28]. The proposed mechanisms include alteration of plaque inflammation, stabilization of vulnerable plaques, and modification of very-low-density lipoprotein and chylomicron metabolism to reduce serum triglyceride levels [29]. A clinical dose of fish oil (4 g/d of EPA and DHA) has been suggested to reduce triglyceride levels [30]. However, it is unknown what dosage level and duration of fish oil are needed to prevent CVD. In addition, the role of omega-3 fats in reducing mortality from CVD has not yet been established. A meta-analysis of 13 randomized controlled trials involving 127,477 participants shows that omega-3 lowers the risk of CVD death, while a Cochrane review including 79 trials involving over 112,000 people shows that increased EPA and DHA level from omega-3 has little or no effect on CVD death, coronary deaths or events, stroke, or heart irregularities [29]. Where these previous studies focused on only fish oil supplements, and other studies established the importance of a healthy diet in CVD and obesity prevention [31,32,33], or the benefits of single food groups (e.g., vegetable) and dietary patterns (e.g., Mediterranean diet) in relation to CVD and obesity [34], remarkably few studies have investigated the combined or joint effects of supplements and dietary quality.

A prominent finding is that combining a healthy diet and calcium supplements is associated with a lower the risk of obesity in middle-aged and older people, which may propose another potential strategy for obesity prevention. Previous studies indicate that obesity often coexists with low calcium intake, suggesting that increased calcium consumption may be a potential strategy to reduce obesity [35,36]. The possible biological mechanisms underlying the effects of calcium on obesity include regulation of adipogenesis, modulation of fat metabolism, promotion of adipocyte proliferation and apoptosis, enhancement of thermogenesis, suppression of fat absorption and promotion of fecal fat excretion, and modification of gut microbiota composition [37]. A meta-analysis including 33 randomized controlled trials and longitudinal studies, with a total of 4733 participants, showed that calcium supplementation reduces body weight in participants who have normal BMI, or in children and adolescents, adult men, or premenopausal women [38]. However, evidence does not consistently support the contention that calcium accelerates weight or fat loss in obese individuals in the general population [35,38]. The evidence of joint effect of diet and calcium supplementation are scarce, indicating further studies are needed.

Our results indicate that the joint effects of diet and dietary supplements in relation to comorbidities may vary across different diseases. The combination of a healthy diet and calcium supplementation was found to lower the risk of obesity, but this association was not found among people with both obesity and CVD. Since comorbidities are increasingly common among older people [39], strong scientific evidence to support the effectiveness of dietary interventions in preventing co-occurring chronic conditions is essential. 

The strengths of our study include comprehensive analyses conducted using longitudinal data from a large population sample, allowing us to track long-term dietary consumption and making an etiological link between diet and supplements in relation to obesity and CVD. However, our study is limited by using self-reported data, including self-reported dietary consumption and health outcomes, which may have measurement bias. Participants who consumed dietary supplements were more likely to have a better diet and be health conscious. For example, participants who consumed multivitamins were more likely to be physically active (82.3% vs. 80.2%, *p* = 0.001) and had a lower likelihood to be excessive alcohol users (23.7% vs. 27.7%, *p* = 0.001) than those not taking multivitamins. These participants may have completed the dietary questionnaire better, leading to higher dietary scores. In addition, it is unknown whether participants only started to take supplements after they had health problems. These may bring potential bias to the results, which may partly explain our results of no joint effect of diet and supplements in relation to CVD and obesity among those with an unhealthy diet. One of the criteria to define CVD was participants that had recent treatment (in the last month) for thrombosis, heart attack or angina, or other types of heart disease. However, it is unknown what types of treatment they have had (e.g., had prescribed medication or CVD intervention procedures). Although detailed dietary assessment such as the Healthy Eating Index may provide insights into dietary quality, we were not able to apply it to our study because the short food frequency questions in the 45 and Up Study do not have detailed information on dietary consumption. For example, the short food frequency questionnaire does not capture all relevant foods, such as yogurt, which was not included in the “dairy” group. Therefore, we generated the dietary score based on the Australia dietary guideline. However, this generated score has limitations. The validation of this score needs to be further examined. Where specific vitamins may have potential benefits, such as vitamin D for CVD and obesity [36,40], our study was unable to distinguish specific vitamins, as they were clustered under multivitamins and minerals in the survey. The questions for calcium intake only included the most commonly used brands. In addition, the questionnaire only provides information about the frequency of food of “grains”, “lean meats, poultry and seafood”, and “dairy”, as well as supplement consumption rather than the exact amount consumed, which prevented us from examining the doses of these food groups and supplement consumption. Participants were only asked whether they had taken supplements in the past 4 weeks. The doses of supplement consumed (e.g., once in 4 weeks or daily in 4 weeks) were unknown and may have under- or overestimated results of the association. However, based on data from other surveys, it is known that most of the dietary supplement users consumed supplements on a daily basis among the Australian population [41].

## 5. Conclusions

Our study results highlight the beneficial role of a long-term healthy diet combined with multivitamins, and minerals or fish oil was associated with a lower incidence of CVD; it also concludes that participants maintained a healthy diet and calcium supplement associated with a lower incidence of obesity. Further large scale and longitudinal epidemiological studies in examining the joint effect of diet and supplements are needed. 

## Figures and Tables

**Table 1:** Participant characteristics at baseline and follow-up.

Variables	Baseline	Follow-Up	*p* Value
	N (%)		
**Socio-demographic variables**			
**Age**			
51–70 years	53,868 (77.0)	38,384 (54.8)	<0.001
>70 years	16,122 (23.0)	31,605 (45.2)	
**Marital status**			
Married/partner	54,454 (78.2)	50,762 (73.2)	<0.001
Single/divorce/separated	10,014 (14.4)	10,135 (14.6)	
Widowed	5166 (7.4)	8480 (12.2)	
SEIFA *			
Low	21,269 (31.3)	23,128 (34.9)	<0.001
Medium	22,951 (33.7)	21,715 (32.8)	
High	23,818 (35.0)	21,291 (32.2)	
**Sex**			
Men	30,325 (43.3)	30,326 (43.3)	NA
Women	39,665 (56.7)	39,665 (56.7)	
**Country of birth**			
Australia	54,828 (78.8)	54,828 (78.8)	NA
Other countries	14,742 (21.2)	14,742 (21.2)	
Education **			
Low	21,910 (31.6)	21,910 (31.6)	NA
Medium	29,098 (42.0)	29,098 (42.0)	
High	18,272 (26.4)	18,272 (26.4)	
**Supplements**			
**Minerals and multivitamins**			
No	51,809 (74.0)	50,513 (72.2)	<0.001
Yes	18,181 (26.0)	19,477 (27.8)	
**Fish oil, omega 3**			
No	42,546 (60.8)	40,103 (57.3)	<0.001
Yes	27,444 (39.2)	29,882 (42.7)	
**Calcium**			
No	62,126 (88.8)	60,733 (86.8)	<0.001
Yes	7863 (11.2)	9254 (13.2)	
**Conditions or disease**			
**Body mass index**			
Underweight	693 (1.0)	893 (1.3)	<0.001
Normal	23,814 (34.0)	21,071 (30.1)	
Overweight	26,627 (38.0)	23,405 (33.4)	
Obesity	18,856 (26.9)	24,621 (35.2)	
**CVD**			
No	59,110 (84.5)	49,534 (70.8)	<0.001
Yes	10,880 (15.6)	20,456 (29.2)	
**Comorbid obesity and CVD**			
No	43,574 (94.4)	32,657 (88.0)	<0.001
Yes	2610 (5.6)	4443 (12.0)	

* Socio-Economic Indexes for Areas (SEIFA) is based on three quantiles (low, medium, high) of Index of Relative Socio-economic Advantage and Disadvantage. ** Education levels: Low: no school certificate or other qualification, and school or intermediate certificate; Medium: high school or leaving certificate, and trade or apprenticeship; and High: certificate or diploma, and university degree or higher.

**Table 2:** The association between dietary or individual supplement consumption and the incidence of obesity, CVD, or both.

	IRR (95% CI) ^¶^			
	Obesity (N = 55,654) *			
Diet	Crude model	Model 2	Model 3	Model 4
Healthy	1.00	1.00	1.00	1.00
Unhealthy	1.06 (1.00; 1.14)	**1.15 (1.08; 1.24)**	**1.09 (1.01; 1.17)**	**1.10 (1.02; 1.18)**
**Minerals and Multivitamins**				
No	1.00	1.00	1.00	1.00
Yes	0.97 (0.91; 1.04)	0.99 (0.92; 1.06)	0.99 (0.93; 1.06)	1.03 (0.96; 1.11)
**Fish oil, omega 3**				
No	1.00	1.00	1.00	1.00
Yes	0.96 (0.91; 1.02)	0.96 (0.90; 1.02)	0.99 (0.93; 1.05)	1.01 (0.95; 1.07)
**Calcium**				
No	1.00	1.00	1.00	1.00
Yes	0.94 (0.86; 1.02)	**0.89 (0.81; 0.98)**	**0.88 (0.80; 0.97)**	**0.83 (0.75; 0.91)**
	CVD (N = 59,110) **			
**Diet**				
Healthy	1.00	1.00	1.00	1.00
Unhealthy	0.98 (0.94; 1.02)	**1.06 (1.02; 1.11)**	1.05 (1.00; 1.10)	1.03 (0.98; 1.08)
**Minerals and Multivitamins**				
No	1.00	1.00	1.00	1.00
Yes	**0.81 (0.77; 0.85)**	**0.92 (0.88; 0.96)**	**0.92 (0.87; 0.96)**	**0.94 (0.89; 0.99)**
**Fish oil, omega 3**				
No	1.00	1.00	1.00	1.00
Yes	**0.89 (0.86; 0.93)**	**0.92 (0.89; 0.96)**	**0.93 (0.88; 0.97)**	**0.95 (0.91; 0.99)**
**Calcium**				
No	1.00	1.00	1.00	1.00
Yes	**1.07 (1.01; 1.13)**	**1.09 (1.02; 1.15)**	1.06 (1.00; 1.13)	1.03 (0.97; 1.11)
	**Obesity and CVD (N = 29,815) ^§^**			
**Diet**				
Healthy	1.00	1.00	1.00	1.00
Unhealthy	1.10 (0.91; 1.33)	1.13 (0.91; 1.39)	1.03 (0.83; 1.28)	0.99 (0.79; 1.24)
**Minerals and Multivitamins**				
No	1.00	1.00	1.00	1.00
Yes	**0.69 (0.55; 0.85)**	**0.78 (0.62; 0.97)**	**0.79 (0.64; 0.99)**	0.84 (0.67; 1.06)
**Fish oil, omega 3**				
No	1.00	1.00	1.00	1.00
Yes	**0.75 (0.63; 0.91)**	**0.80 (0.66; 0.98)**	0.85 (0.70; 1.04)	0.87 (0.71; 1.07)
**Calcium**				
No	1.00	1.00	1.00	1.00
Yes	0.94 (0.72; 1.23)	1.00 (0.74; 1.34)	0.99 (0.74; 1.34)	0.89 (0.66; 1.21)

^¶^ Bold: *p* < 0.05. * Model 2 after adjustment of age, gender, country of birth, marital status, education, SEIFA; model 3 after adjustment of smoking, physical activity, and model 2; model 4 after adjustment of CVD, diabetes, blood cholesterol, blood pressure, and model 3. ** Model 2 after adjustment of age, gender, country of birth, marital status, education, and SEIFA; model 3 after adjustment of smoking, physical activity, and model 2; model 4 after adjustment of obesity, diabetes, blood cholesterol, blood pressure, and obesity. ^§^ Model 2 after adjustment of age, gender, country of birth, marital status, education, and SEIFA; model 3 after adjustment of smoking, physical activity, and model 2; model 4 after diabetes, blood cholesterol, blood pressure, and model 3.

**Table 3:** The joint effects of healthy vs. unhealthy diet and dietary supplements in relation to the incidence of both obesity and CVD (**N = 29,815**).

	Incidence of People with Obesity and CVD *			
	IRR (95% CI) ^¶^			
	Crude Model	Model 2	Model 3	Model 4
**Minerals and multivitamins (N = 29,813)**				
Healthy diet (+), no MVM (–)	1.00	1.00	1.00	1.00
Healthy diet (+), new MVM (+)	0.88 (0.64; 1.23)	1.05 (0.75; 1.46)	1.07 (0.77; 1.50)	1.14 (0.82; 1.59)
Healthy diet (+), former MVM (+, –)	1.11 (0.80; 1.53)	1.23 (0.87; 1.74)	1.20 (0.84; 1.71)	1.28 (0.90; 1.82)
Healthy diet (+), long-term MVM (+, +)	**0.49 (0.34; 0.71)**	**0.55 (0.37; 0.81)**	**0.60 (0.38; 0.83)**	**0.61 (0.41; 0.91)**
Unhealthy diet (–), No MVM (–)	1.11 (0.88; 1.41)	1.14 (0.88; 1.47)	1.04 (0.80; 1.35)	1.00 (0.77; 1.31)
Unhealthy diet (–), new MVM (+)	0.88 (0.54; 1.45)	0.97 (0.56; 1.67)	0.85 (0.48; 1.49)	0.81 (0.44; 1.48)
Unhealthy diet (–), former MVM (+, –)	1.11 (0.68; 1.81)	1.31 (0.79; 2.18)	1.22 (0.73; 2.04)	1.31 (0.78; 2.20)
Unhealthy diet (–), long-term MVM (+, +)	0.62 (0.38; 1.03)	0.78 (0.46; 1.32)	0.74 (0.44; 1.26)	0.78 (0.45; 1.35)
**Fish oil (N = 29,814)**				
Healthy diet (+), no fish oil (–)	1.00	1.00	1.00	1.00
Healthy diet (+), new fish oil (+)	**0.68 (0.49; 0.94)**	**0.70 (0.50; 0.98)**	0.74 (0.53; 1.05)	0.80 (0.57; 1.13)
Healthy diet (+), former fish oil (+, –)	0.98 (0.71; 1.35)	0.99 (0.71; 1.39)	1.06 (0.75; 1.49)	1.09 (0.77; 1.53)
Healthy diet (+), long-term fish oil (+, +)	**0.64 (0.49; 0.84)**	**0.68 (0.51; 0.90)**	**0.72 (0.54; 0.96)**	0.78 (0.58; 1.03)
Unhealthy diet (–), no fish oil (–)	1.06 (0.81; 1.37)	1.05 (0.79; 1.40)	0.96 (0.72; 1.29)	0.95 (0.71; 1.28)
Unhealthy diet (–), new fish oil (+)	0.91 (0.60; 1.38)	1.08 (0.70; 1.66)	1.06 (0.68; 1.64)	1.07 (0.68; 1.68)
Unhealthy diet (–), former fish oil (+, –)	0.85 (0.51; 1.41)	0.85 (0.48; 1.51)	0.82 (0.47; 1.45)	0.88 (0.50; 1.56)
Unhealthy diet (–), long–term fish oil (+, +)	**0.63 (0.40; 0.98)**	0.73 (0.47; 1.16)	0.73 (0.46; 1.15)	0.70 (0.43; 1.14)
**Calcium (N = 29,814)**				
Healthy diet (+), no calcium (–)	1.00	1.00	1.00	1.00
Healthy diet (+), new calcium (+)	0.91 (0.60; 1.36)	0.96 (0.62; 1.47)	0.94 (0.61; 1.47)	0.85 (0.55; 1.33)
Healthy diet (+), former calcium (+, –)	0.58 (0.33; 1.04)	0.68 (0.38; 1.22)	0.71 (0.39; 1.27)	0.69 (0.38; 1.23)
Healthy diet (+), long-term calcium (+, +)	0.78 (0.50; 1.20)	0.76 (0.48; 1.22)	0.80 (0.50; 1.27)	0.72 (0.45; 1.14)
Unhealthy diet (–), no calcium (–)	1.08 (0.88; 1.33)	1.11 (0.89; 1.39)	1.03 (0.81; 1.29)	0.99 (0.78; 1.26)
Unhealthy diet (–), new calcium (+)	1.18 (0.63; 2.22)	1.37 (0.71; 2.66)	1.14 (0.56; 2.30)	1.03 (0.49; 2.17)
Unhealthy diet (–), former calcium (+, –)	0.60 (0.19; 1.88)	0.55 (0.14; 2.19)	0.51 (0.13; 2.05)	0.51 (0.13; 2.06)
Unhealthy diet (–), long-term calcium (+, +)	0.61 (0.23; 1.65)	0.80 (0.30; 2.14)	0.74 (0.24; 1.24)	0.71 (0.27; 1.90)

* Model 2 after adjustment of age, gender, country of birth, marital status, education, and SEIFA; model 3 after adjustment of smoking, physical activity, and model 2; model 4 after diabetes, blood cholesterol, blood pressure, and model 2. ^¶^ Bold: *p* < 0.05.

## Data Availability

The 45 and Up Study is managed by the Sax Institute. For data access, please contact the 45 and Up Study team at 45 and Up.research@saxinstitute.org.au.

## Supplementary Materials

The following are available online at https://www.mdpi.com/2072-6643/13/3/944/s1, Table S1. Scoring dietary consumption; Table S2. Group classifications of joint diet and dietary supplements consumption; Table S3. The joint effects of healthy vs. unhealthy diet and dietary supplements in relation to the incidence of both obesity and CVD (N = 69,990); Table S4. The joint effects of healthy vs. unhealthy diet and dietary supplements in relation to the incidence of both obesity and CVD (N = 100,109); Figure S1. The joint effects of healthy vs. unhealthy diet and minerals and multivitamins consumption, and the incidence of obesity and CVD (N = 69,990); Figure S2. The joint effects of healthy vs. unhealthy diet and fish oil consumption in relation to the incidence of obesity and CVD (N = 69,990); Figure S3. The joint effects of healthy vs. unhealthy diet and calcium consumption in relation to the incidence of obesity and CVD (N = 69,990); Figure S4. The joint effects of healthy vs. unhealthy diet and minerals and multivitamins consumption and the incidence of obesity and CVD (N = 100,109); Figure S5. The joint effects of healthy vs. unhealthy diet and fish oil consumption in relation to the incidence of obesity and CVD (N = 100,109); Figure S6. The joint effects of healthy vs. unhealthy diet and calcium consumption in relation to the incidence of obesity and CVD (N = 100,109).
