# Supplementary material for: The Joint Effects of Diet and Dietary Supplements in Relation to Obesity and Cardiovascular Disease over a 10-Year Follow-Up: A Longitudinal Study of 69,990 Participants in Australia
Source: Nutrients. 2021 Mar 15;13(3):944. doi: 10.3390/nu13030944 (PMC8001791; doi:10.3390/nu13030944)
Supplement: Supplementary file 1 [file nutrients-13-00944-s001.pdf]

Table S1. Scoring dietary consumption

Dietary consumption	Men Age groups		Women Age groups	
	**51-70 years**	**70+ years**	**51-70 years**	**70+ years**
Vegetable[¶]	≥5.5 serve per day=0<br><5.5 serve per day=1	≥5 serve per day=0<br><5 serve per day=1	≥5 serve per day=0<br><5 serve per day=1	≥5 serve per day=0<br><5 serve per day=1
Fruit[¶]	≥2 serve per day=0<br><2 serve per day=1			
Grains	High frequency=0<br>Medium frequency=1<br>Low frequency=2			
Lean meats, poultry and seafood*	High frequency=0<br>Medium frequency=1<br>Low frequency=2			
Dairy**	Yes=0<br>No=1			
Food diversity	Consume all five groups=0, Not consume all five groups=1			
Alcohol consumption	≤10 standard drink per week=0, >10 standard drink per week=1			

[¶] A serve of vegetable was considered as half a cup of cooked vegetables or one cup of salad. A serve of fruit was considered as 1 medium piece or 2 small pieces or1 cup of diced or canned fruit pieces.

** include beef, lamb, pork, chicken, turkey, duck, fish, and seafood.*

*** include cheese and milk*

Table S2. Group classifications of joint diet and dietary supplements consumption

Group classifications	N	Explanations
**MVM**		
Healthy diet (+), no MVM (-)	31,058	Dietary behavior scores less than mean (0-4), had no MVM at baseline and follow-up
Healthy diet (+), new MVM (+)	6,223	Dietary behavior scores less than mean (0-4), had no MVM at baseline, but had MVM at follow-up
Healthy diet (+), former MVM (+, -)	5,317	Dietary behavior scores less than mean (0-4), had MVM at baseline, but no MVM at follow-up
Healthy diet (+), long-term MVM (+, +)	7,967	Dietary behavior scores less than mean (0-4), had MVM at baseline and follow-up
Unhealthy diet (-), no MVM (-)	12,134	Dietary behavior scores greater than mean (5-9), had no MVM at baseline and follow-up
Unhealthy diet (-), new MVM (+)	2,390	Dietary behavior scores greater than mean (5-9), had no MVM at baseline, but had MVM at follow-up
Unhealthy diet (-), former MVM (+, -)	2,000	Dietary behavior scores greater than mean (5-9), had MVM at baseline, but no MVM at follow-up
Unhealthy diet (-), long-term MVM (+, +)	2,897	Dietary behavior scores greater than mean (5-9), had MVM at baseline and follow-up
**Fish oil**		
Healthy diet (+), no fish oil (-)	21,179	Dietary behavior scores less than mean (0-4), had no fish oil at baseline and follow-up
Healthy diet (+), new fish oil (+)	8,155	Dietary behavior scores less than mean (0-4), had no fish oil at baseline, but had fish oil at follow-up
Healthy diet (+), former fish oil (+, -)	6,534	Dietary behavior scores less than mean (0-4), had fish oil at baseline, but no fish oil at follow-up
Healthy diet (+), long-term fish oil (+, +)	14,696	Dietary behavior scores less than mean (0-4), had fish oil at baseline and follow-up
Unhealthy diet (-), no fish oil (-)	10,104	Dietary behavior scores greater than mean (5-9), had no fish oil at baseline and follow-up
Unhealthy diet (-), new fish oil (+)	3,105	Dietary behavior scores greater than mean (5-9), had no fish oil at baseline, but had fish oil at follow-up
Unhealthy diet (-), former fish oil (+, -)	2,286	Dietary behavior scores greater than mean (5-9), had fish oil at baseline, but no fish oil at follow-up
Unhealthy diet (-), long-term fish oil (+, +)	3,926	Dietary behavior scores greater than mean (5-9), had fish oil at baseline and follow-up
**Calcium**		
Healthy diet (+), no Calcium (-)	40,414	Dietary behavior scores less than mean (0-4), had no Calcium at baseline and follow-up
Healthy diet (+), new Calcium (+)	3,755	Dietary behavior scores less than mean (0-4), had no Calcium at baseline, but had Calcium at follow-up
Healthy diet (+), former Calcium (+, -)	2,776	Dietary behavior scores less than mean (0-4), had Calcium at baseline, but no Calcium at follow-up
Healthy diet (+), long-term Calcium (+, +)	3,620	Dietary behavior scores less than mean (0-4), had Calcium at baseline and follow-up
Unhealthy diet (-), no Calcium (-)	16,856	Dietary behavior scores greater than mean (5-9), had no Calcium at baseline and follow-up
Unhealthy diet (-), new Calcium (+)	1,099	Dietary behavior scores greater than mean (5-9), had no Calcium at baseline, but had Calcium at follow-up
Unhealthy diet (-), former Calcium (+, -)	687	Dietary behavior scores greater than mean (5-9), had Calcium at baseline, but no Calcium at follow-up
Unhealthy diet (-), long-term Calcium (+, +)	779	Dietary behavior scores greater than mean (5-9), had Calcium at baseline and follow-up

Figure S1. The joint effects of healthy vs. unhealthy diet and minerals and multivitamins consumption and the incidence of obesity and CVD (N=69,990)*

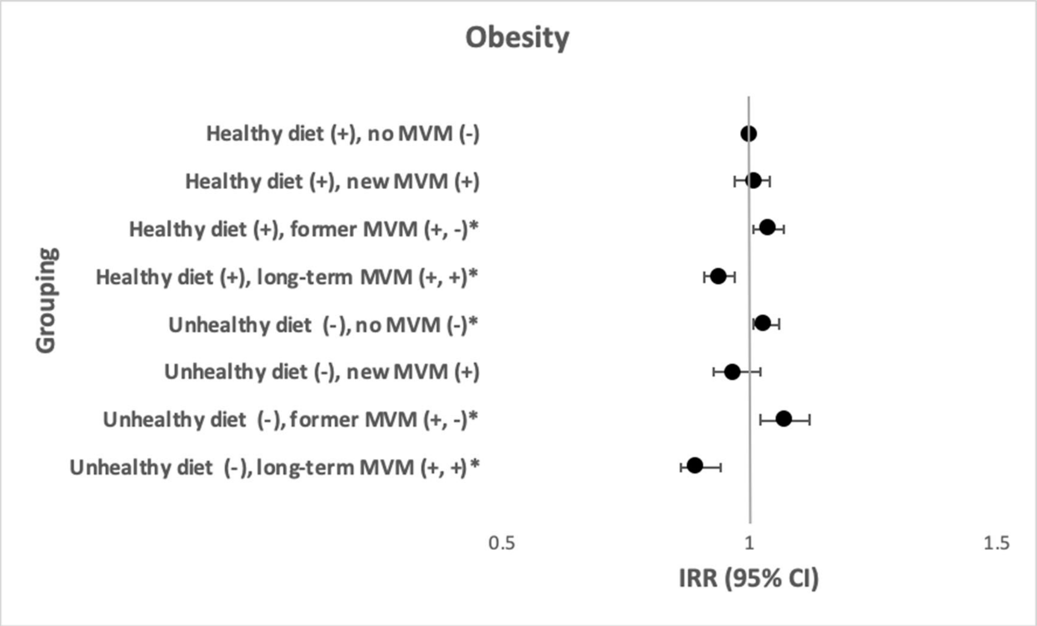

**IRR after adjustment of age, gender, country of birth, marital status, education, SEIFA, smoking, physical activity, CVD, diabetes, blood cholesterol and blood pressure.*

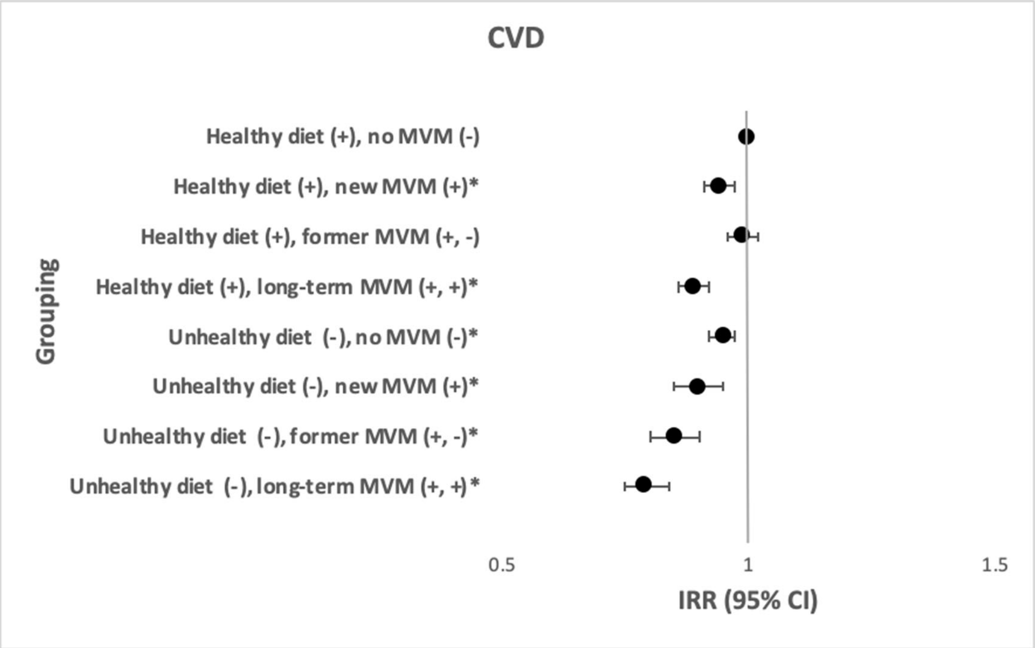

**IRR after adjustment of age, gender, country of birth, marital status, education, SEIFA, smoking, physical activity, obesity, diabetes, blood cholesterol and blood pressure.*

Figure S2. The joint effects of healthy vs. unhealthy diet and fish oil consumption in relation to the incidence of obesity and CVD (N=69,990)*

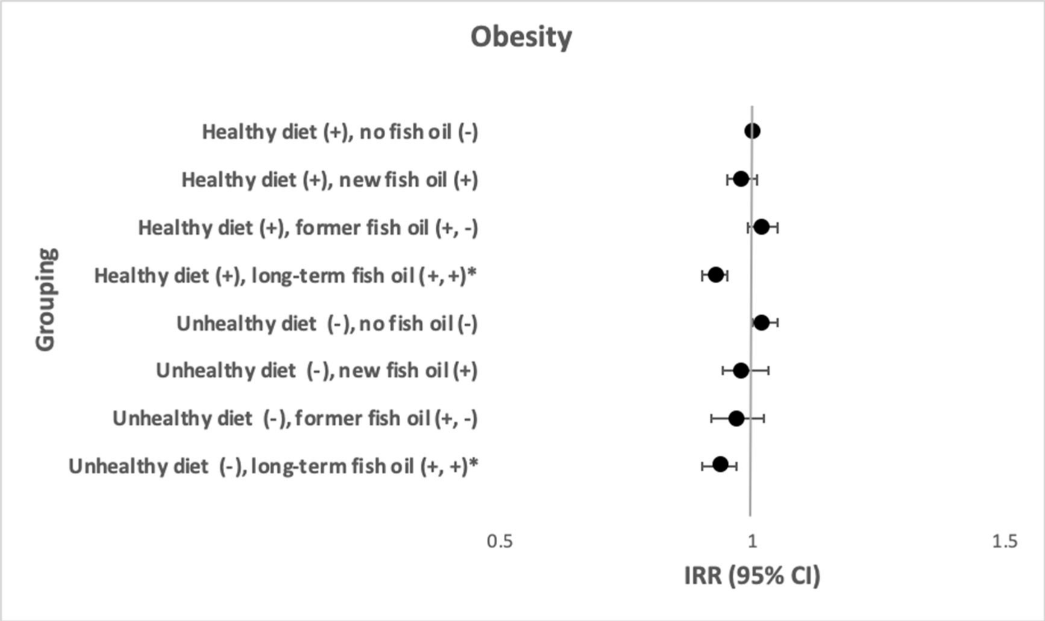

IRR after adjustment of age, gender, country of birth, marital status, education, SEIFA, smoking, physical activity, CVD, diabetes, blood cholesterol and blood pressure.

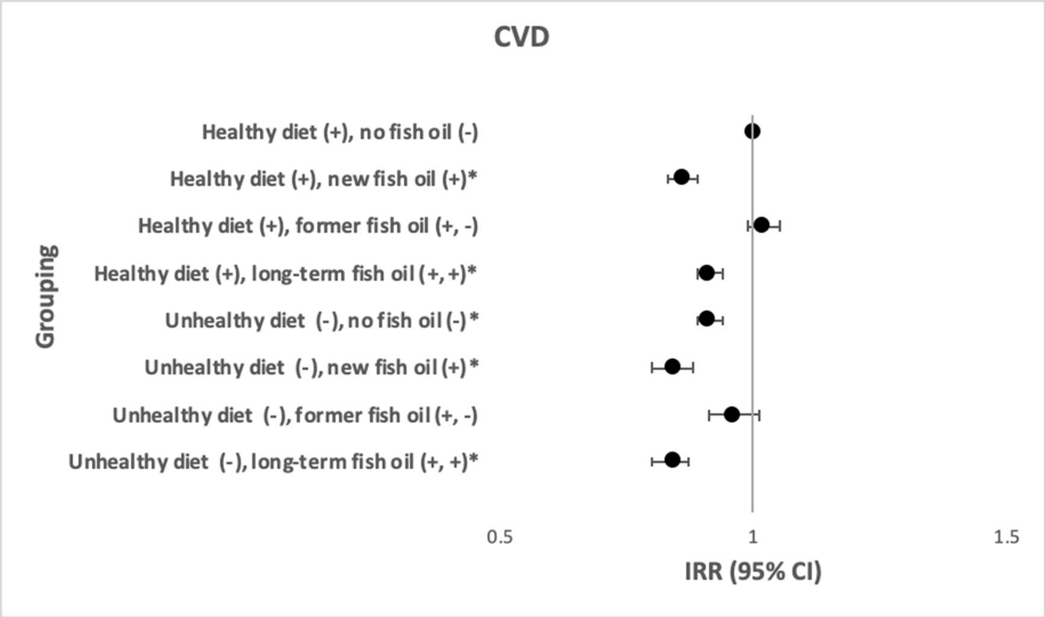

IRR after adjustment of age, gender, country of birth, marital status, education, SEIFA, smoking, physical activity, obesity, diabetes, blood cholesterol and blood pressure.

Figure S3. The joint effects of healthy vs. unhealthy diet and calcium consumption in relation to the incidence of obesity and CVD (N=69,990)*

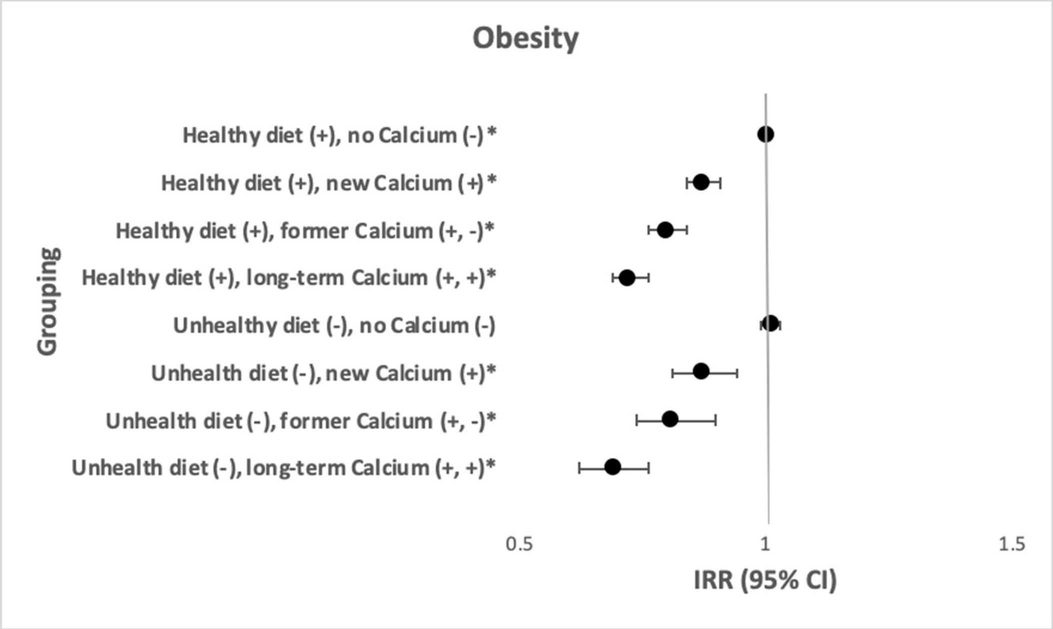

**IRR after adjustment of age, gender, country of birth, marital status, education, SEIFA, smoking, physical activity, CVD, diabetes, blood cholesterol, blood pressure and osteoarthritis.*

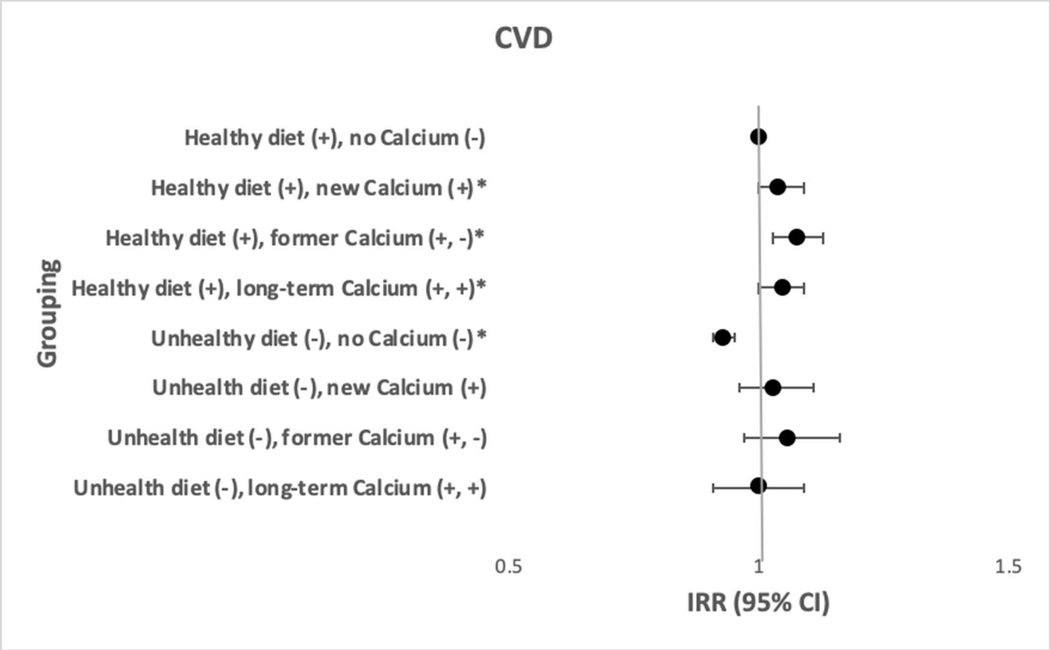

**IRR after adjustment of age, gender, country of birth, marital status, education, SEIFA, smoking, physical activity, obesity, diabetes, blood cholesterol, blood pressure and osteoarthritis.*

Table S3. The joint effects of healthy vs. unhealthy diet and dietary supplements in relation to the incidence of both obesity and CVD (N=69,990)

	Incidence of people with obesity and CVD* IRR (95% CI) ¶			
	**Crude model**	**Model 2**	**Model 3**	**Model 4**
**MVM**				
Healthy diet (+), no MVM (-)	1.00	1.00	1.00	1.00
Healthy diet (+), new MVM (+)	**0.87 (0.82; 0.93)**	**0.93 (0.87; 0.99)**	0.94 (0.88; 1.00)	0.97 (0.91; 1.03)
Healthy diet (+), former MVM (+, -)	**0.89 (0.83; 0.95)**	0.97 (0.90; 1.04)	0.96 (0.89; 1.03)	1.01 (0.94; 1.08)
Healthy diet (+), long-term MVM (+, +)	**0.70 (0.65; 0.74)**	**0.77 (0.72; 0.82)**	**0.78 (0.72; 0.83)**	**0.85 (0.80; 0.91)**
Unhealthy diet (-), No MVM (-)	1.04 (0.99; 1.09)	1.05 (1.00; 1.10)	0.96 (0.91; 1.00)	0.97 (0.92; 1.02)
Unhealthy diet (-), new MVM (+)	**0.85 (0.77; 0.94)**	0.92 (0.83; 1.01)	**0.84 (0.75; 0.93)**	0.91 (0.82; 1.00)
Unhealthy diet (-), former MVM (+, -)	**0.80 (0.71; 0.90)**	0.89 (0.79; 1.00)	**0.82 (0.72; 0.92)**	**0.88 (0.78; 0.99)**
Unhealthy diet (-), long-term MVM (+, +)	**0.64 (0.58; 0.71)**	**0.70 (0.63; 0.78)**	**0.66 (0.59; 0.73)**	**0.72 (0.65; 0.80)**
**Fish oil**				
Healthy diet (+), no fish oil (-)	1.00	1.00	1.00	1.00
Healthy diet (+), new fish oil (+)	**0.77 (0.72; 0.82)**	**0.80 (0.75; 0.85)**	**0.81 (0.76; 0.87)**	**0.87 (0.82; 0.93)**
Healthy diet (+), former fish oil (+, -)	1.00 (0.94; 1.07)	1.02 (0.96; 1.08)	1.03 (0.97; 1.10)	1.04 (0.98; 1.11)
Healthy diet (+), long-term fish oil (+, +)	**0.73 (0.69; 0.77)**	**0.77 (0.73; 0.81)**	**0.80 (0.76; 0.85)**	**0.86 (0.81; 0.90)**
Unhealthy diet (-), no fish oil (-)	0.99 (0.94; 1.04)	0.99 (0.94; 1.04)	**0.90 (0.85; 0.95)**	**0.93 (0.88; 0.98)**
Unhealthy diet (-), new fish oil (+)	**0.77 (0.71; 0.85)**	**0.82 (0.74; 0.90)**	**0.79 (0.72; 0.87)**	**0.86 (0.78; 0.94)**
Unhealthy diet (-), former fish oil (+, -)	**0.90 (0.81; 0.99)**	0.95 (0.86; 1.06)	0.90 (0.81; 1.00)	0.95 (0.86; 1.04)
Unhealthy diet (-), long-term fish oil (+, +)	**0.72 (0.66; 0.78)**	**0.77 (0.70; 0.84)**	**0.74 (0.67; 0.80)**	**0.79 (0.72; 0.86)**
**Calcium**				
Healthy diet (+), no Calcium (-)	1.00	1.00	1.00	1.00
Healthy diet (+), new Calcium (+)	**0.85 (0.78; 0.92)**	**0.88 (0.81; 0.96)**	**0.89 (0.82; 0.97)**	**0.86 (0.80; 0.94)**
Healthy diet (+), former Calcium (+, -)	**0.81 (0.73; 0.89)**	**0.88 (0.80; 0.98)**	**0.88 (0.79; 0.97)**	**0.89 (0.80; 0.98)**
Healthy diet (+), long-term Calcium (+, +)	**0.73 (0.67; 0.80)**	**0.78 (0.71; 0.85)**	**0.79 (0.72; 0.86)**	**0.77 (0.70; 0.84)**
Unhealthy diet (-), no Calcium (-)	0.99 (0.95; 1.03)	1.00 (0.96; 1.04)	**0.92 (0.88; 0.96)**	**0.94 (0.90; 0.98)**
Unhealthy diet (-), new Calcium (+)	0.92 (0.80; 1.05)	1.05 (0.91; 1.20)	0.95 (0.83; 1.10)	0.99 (0.86; 1.13)
Unhealthy diet (-), former Calcium (+, -)	**0.66 (0.53; 0.82)**	**0.76 (0.61; 0.96)**	**0.70 (0.55; 0.87)**	**0.72 (0.58; 0.90)**
Unhealthy diet (-), long-term Calcium (+, +)	**0.66 (0.54; 0.80)**	**0.76 (0.63; 0.93)**	**0.70 (0.57; 0.85)**	**0.70 (0.58; 0.85)**

** Model 1 is the crude model; model 2 after adjustment of age, gender, country of birth, marital status, education, SEIFA; model 3 after adjustment of smoking, physical activity; model 4 after diabetes, blood cholesterol and blood pressure. For calcium supplementation, model 4 further adjusted for osteoarthritis. ¶ Bold: $p<0.05$.*

Figure S4. The joint effects of healthy vs. unhealthy diet and minerals and multivitamins consumption and the incidence of obesity and CVD (N=100,109)*

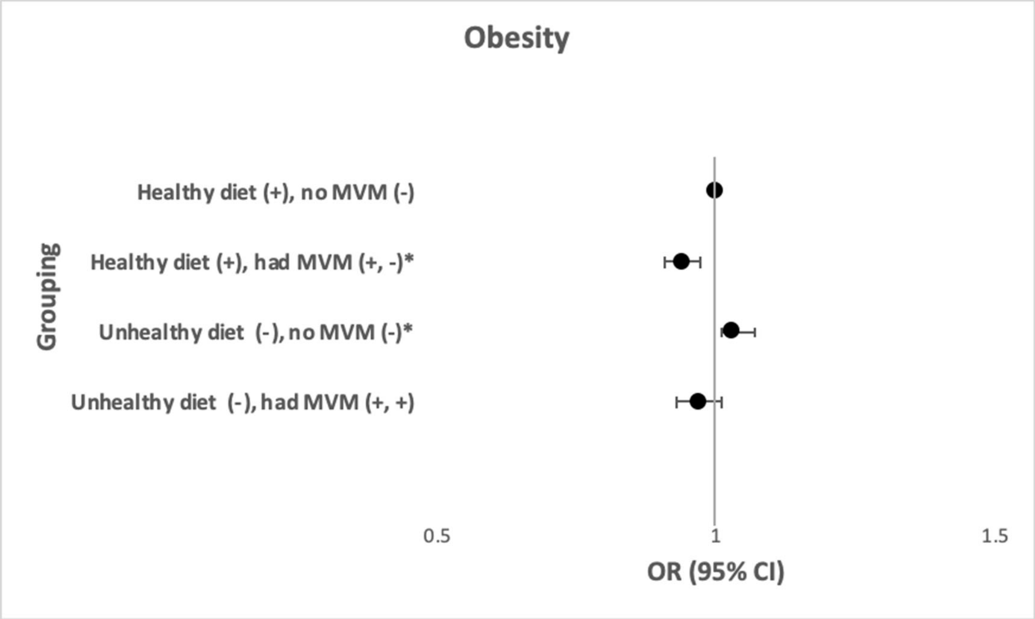

**OR after adjustment of age, gender, country of birth, marital status, education, SEIFA, smoking, physical activity, CVD, diabetes, blood cholesterol, blood pressure and survey wave.*

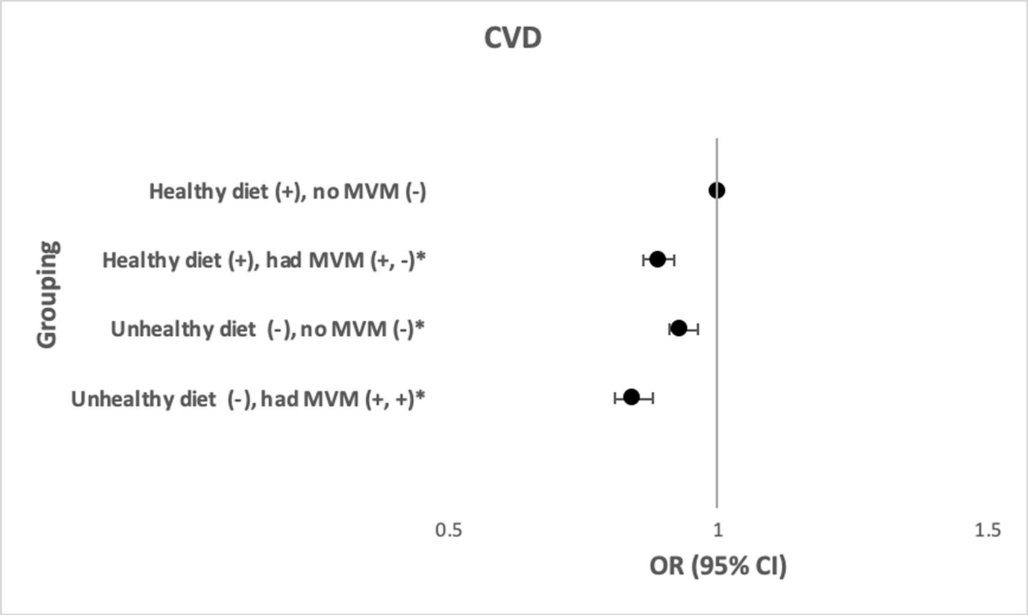

**OR after adjustment of age, gender, country of birth, marital status, education, SEIFA, smoking, physical activity, obesity, diabetes, blood cholesterol, blood pressure and survey wave.*

Figure S5. The joint effects of healthy vs. unhealthy diet and fish oil consumption in relation to the incidence of obesity and CVD (N=100,109)*

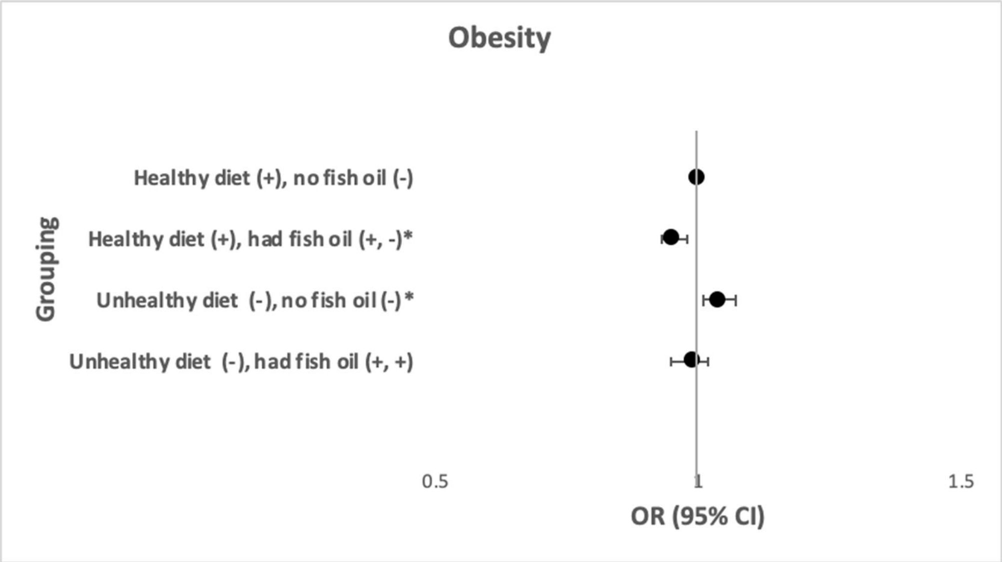

**OR after adjustment of age, gender, country of birth, marital status, education, SEIFA, smoking, physical activity, CVD, diabetes, blood cholesterol, blood pressure and survey wave.*

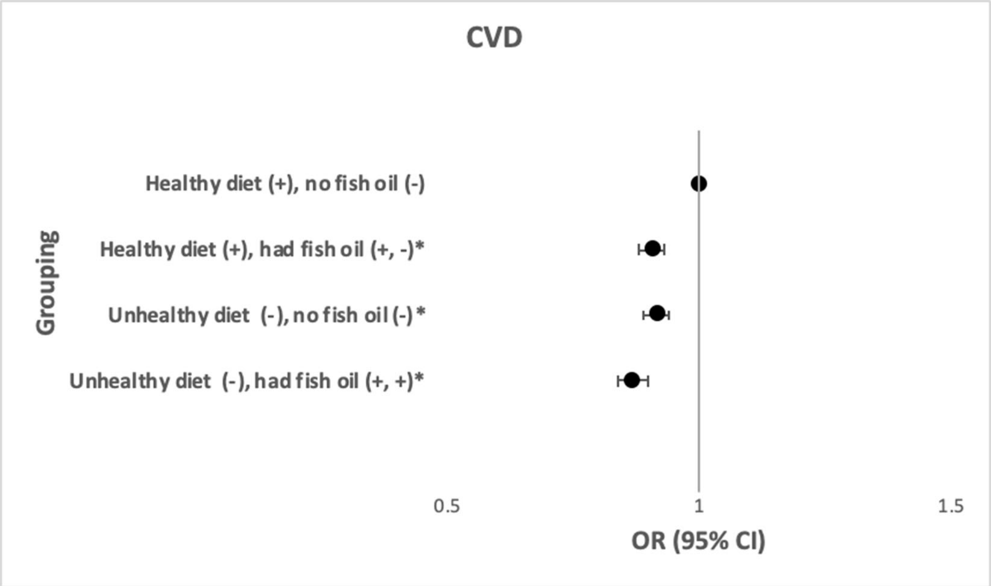

**OR after adjustment of age, gender, country of birth, marital status, education, SEIFA, smoking, physical activity, obesity, diabetes, blood cholesterol, blood pressure and survey wave.*

Figure S6. The joint effects of healthy vs. unhealthy diet and calcium consumption in relation to the incidence of obesity and CVD (N=100,109)*

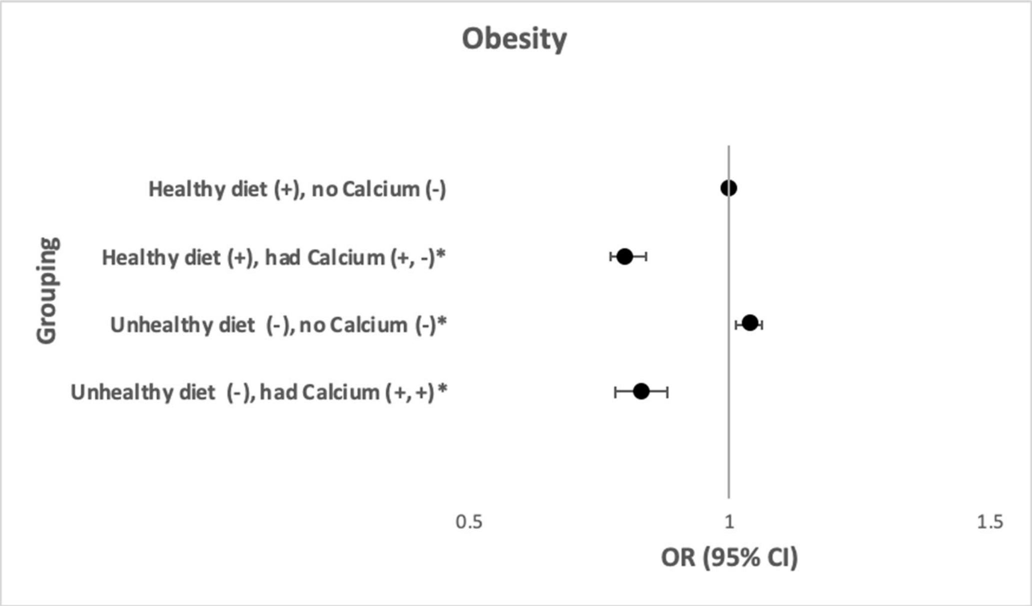

**OR after adjustment of age, gender, country of birth, marital status, education, SEIFA, smoking, physical activity, CVD, diabetes, blood cholesterol, blood pressure, osteoarthritis and survey wave.*

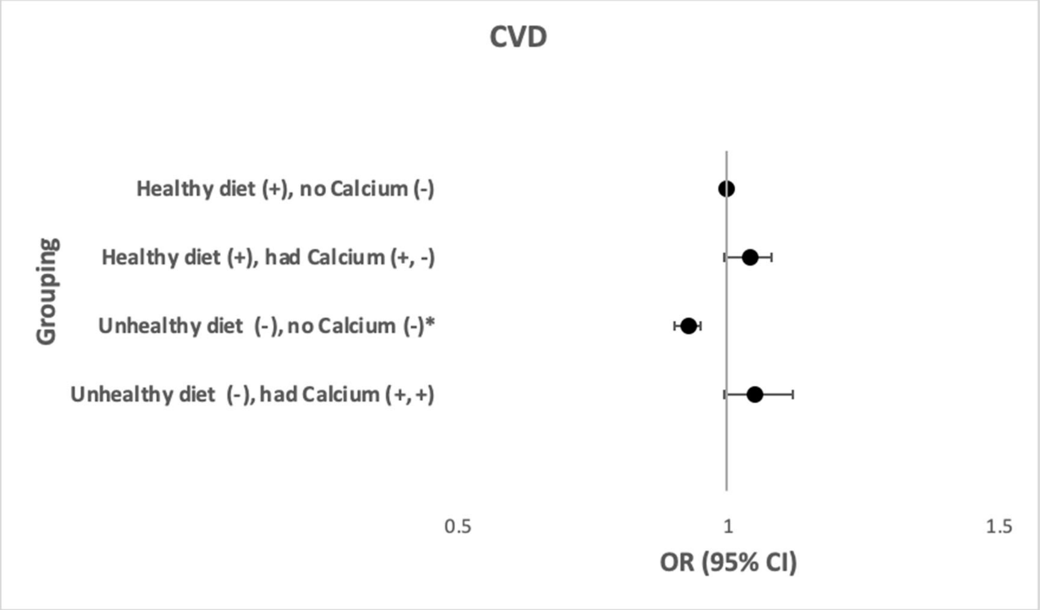

**OR after adjustment of age, gender, country of birth, marital status, education, SEIFA, smoking, physical activity, obesity, diabetes, blood cholesterol, blood pressure, osteoarthritis and survey wave.*

Table S4. The joint effects of healthy vs. unhealthy diet and dietary supplements in relation to the incidence of both obesity and CVD (N=100,109)

	Comorbid obesity in people with CVD* Odds Ratio (95% CI) ¶			
	**Model 1**	**Model 2**	**Model 3**	**Model 4**
**MVM**				
Healthy diet (+), no MVM (-)	1.00	1.00	1.00	1.00
Health diet (+), had MVM (+)	**0.84 (0.80; 0.88)**	**0.90 (0.85; 0.94)**	**0.90 (0.85; 0.95)**	**0.91 (0.86; 0.97)**
Unhealthy diet (-), no MVM (-)	1.04 (0.99; 1.08)	1.04 (0.99; 1.09)	1.00 (0.95; 1.04)	1.01 (0.96; 1.06)
Unhealth diet (-), had MVM (+)	**0.86 (0.81; 0.92)**	**0.91 (0.85; 0.97)**	**0.88 (0.82; 0.94)**	**0.88 (0.82; 0.95)**
**Fish oil**				
Healthy diet (+), no fish oil (-)	1.00	1.00	1.00	1.00
Health diet (+), had fish oil (+)	**0.85 (0.81; 0.89)**	**0.87 (0.83; 0.91)**	**0.88 (0.84; 0.93)**	**0.88 (0.83; 0.92)**
Unhealthy diet (-), no fish oil (-)	1.00 (0.96; 1.04)	1.00 (0.96; 1.04)	0.96 (0.92; 1.01)	0.98 (0.93; 1.03)
Unhealth diet (-), had fish oil (+)	**0.92 (0.87; 0.98)**	**0.94 (0.89; 1.00)**	**0.92 (0.87; 0.98)**	**0.90 (0.84; 0.96)**
**Calcium**				
Healthy diet (+), no Calcium (-)	1.00	1.00	1.00	1.00
Health diet (+), had Calcium (+)	**0.89 (0.83; 0.95)**	**0.91 (0.85; 0.98)**	**0.91 (0.84; 0.98)**	**0.84 (0.78; 0.91)**
Unhealthy diet (-), no Calcium (-)	1.03 (0.99; 1.07)	1.03 (0.99; 1.07)	0.99 (0.95; 1.03)	0.99 (0.95; 1.04)
Unhealth diet (-), had Calcium (+)	0.96 (0.87; 1.05)	1.01 (0.92; 1.12)	0.97 (0.87; 1.08)	**0.86 (0.77; 0.97)**

** Model 1 is the crude model; model 2 after adjustment of age, gender, country of birth, marital status, education, SEIFA; model 3 after adjustment of smoking, physical activity; model 4 after wave, diabetes, blood cholesterol and blood pressure. For calcium supplementation, model 4 further adjusted for osteoarthritis.*

¶*Bold: p<0.05.*
